# “Alterations in the Skin Microbiota Are Associated With Symptom Severity in Mycosis Fungoides”

**DOI:** 10.3389/fcimb.2022.850509

**Published:** 2022-05-17

**Authors:** Yumeng Zhang, Lucia Seminario-Vidal, Leah Cohen, Mohammad Hussaini, Jiqiang Yao, David Rutenberg, Youngchul Kim, Anna Giualiano, Lary A. Robinson, Lubomir Sokol

**Affiliations:** ^1^ Department of Medicine, University of South Florida, Tampa, FL, United States; ^2^ Department of Malignant Hematology, Moffitt Cancer Center, Tampa, FL, United States; ^3^ Department of Cutaneous Oncology, Moffitt Cancer Center, Tampa, FL, United States; ^4^ Department of Dermatology, University of South Florida, Tampa, FL, United States; ^5^ Department of Dermatology, University of Pennsylvania, Philadelphia, PA, United States; ^6^ Department of Pathology, Moffitt Cancer Center, Tampa, FL, United States; ^7^ Department of Biostatistics and Bioinformatics, Moffitt Cancer Center, Tampa, FL, United States; ^8^ Department of Cancer Epidemiology, Moffitt Cancer Center, Tampa, FL, United States; ^9^ Department of Thoracic Oncology, Moffitt Cancer Center, Tampa, FL, United States

**Keywords:** mycosis fundgoides, cutaneous T cell lymphoma, skin microbiota, microbiome and dysbiosis, disease phenotype association

## Abstract

Cutaneous T cell lymphoma (CTCL), a non-Hodgkin lymphoma, is thought to arise from mature tissue-resident memory T cells. The most common subtypes include Mycosis Fungoides and Sezary Syndrome. The role of skin microbiota remains unclear in the symptom manifestation of MF. Among 39 patients with MF, we analyzed bacteria colonizing MF lesions and non-lesional skin in the contralateral side and characterized regional changes in the skin microbiota related to MF involvement using the difference in relative abundance of each genus between lesional and contralateral non-lesional skin. We investigated the relationship between these skin microbiota alterations and symptom severity. No statistically significant difference was found in bacterial diversity and richness between lesional and non-lesional skin. Different skin microbiota signatures were associated with different symptoms. More pronounced erythema in the lesions was associated with an increase in *Staphylococcus*. Pain and thick skin in the lesions were associated with a decrease in *Propionibacterium*. The results of this pilot study suggest that the skin microbiota plays an important role in changing skin phenotypes among patients with MF. Larger skin microbiota studies are needed to confirm these findings and support the use of antibiotic treatment to mitigate CTCL symptoms.

## Introduction

Cutaneous T cell lymphoma (CTCL), a type of non-Hodgkin lymphoma, is thought to derive from mature tissue-resident memory T cells. Mycosis fungoides (MF) and Sézary syndrome compose about 60% of all CTCLs. Currently, it’s unclear how the symptom manifestation of CTCL is affected by the skin microbiota.

Recently, *Staphylococcus aureus* colonization of cutaneous lesions has been implicated in the pathogenesis of CTCL (Tokura et al., 1995, Tokura et al., 1992, Blumel et al., 2020). *In vitro* studies have highlighted several mechanisms by which staphylococcal endotoxin promotes development of lymphoma (Tokura et al., 1995, Jackow et al., 1997). This endotoxin can act as a superantigen to activate T cells by binding directly to major histocompatibility complex class II molecules, causing oligoclonal T cell receptor Vβ gene expansion. *S. aureus* alpha toxin also inhibits cytotoxic killing of CTCL cells by CD8+ T cells and positively selects for malignant T cells (Blumel et al., 2020). *S. aureus* enterotoxin stimulates nonmalignant T cells to induce *FOXP3* expression and CD25 upregulation in malignant T cells (Woetmann et al., 2007, Willerslev-Olsen et al., 2020). In addition, *S. aureus* colonization can potentially break down skin barrier function and increase antigen exposure on the skin (Nowicka and Grywalska, 2018). Clinically, reduction in disease severity with sustained responses was reported in 8 patients with advanced CTCL for whom broad-spectrum intravenous antibiotics (carbapenem) were used (Lindahl et al., 2019).

We performed a prospective pilot study to characterize the skin microbiota changes among patients with MF. To understand if the skin microbiota affects disease phenotype, we investigated the relationship between the severity of symptoms/signs and skin microbiota alterations.

## Results

### Participant Characteristics

Forty patients with MF consented to participate. One patient was excluded from the study due to change of diagnosis to eczema after histopathologic review. Among 39 included patients, 23 (59%) patients were men, the median age at diagnosis was 64 years (range, 32-77 years), and 32 (82%) were white. Seventeen patients (44%) had a history of previous smoking, but there were no current smokers (**Table 1**). At the time of diagnosis, 30 patients (77%) had patch-stage/plaque-stage disease, and 5 (13%) had tumor-stage disease. Four patients (10%) had erythroderma at the time of diagnosis but did not meet the criteria for Sezary Syndrome due to lack of blood or visceral involvement. The photographs of skin lesions, the percentage of involved total body surface area, and the modified Severity-Weighted Assessment Tool (mSWAT) score at the time of collection are presented in **Figure S1**.

**Table 1:** Patient characteristics.

Parameter	Patients with Mycosis Fungoides (n = 39)
Male sex, n (%)	23 (59)
Race, n (%)	
White	32 (82)
Black	7 (18)
Age, median (range), years	64 (32-77)
Previous smoker, n (%)	17 (44)
Disease stage at diagnosis, n (%)	
Early stage (I-IIA)	29 (74)
Advanced stage (IIB-IV)	10 (26)
Skin T stage at diagnosis, n (%)	
Patch/Plague (I-II)	30 (77)
Tumors (III)	5 (13)
Erythroderma (IV)	4 (10)
Anatomical sites sampled, n (%)	
Trunk	14 (36)
Buttock	7 (18)
Extremities	14 (36)
Head and neck	4 (10)
Current treatment information, n (%)^1^	
No treatment	12 (31)
Topical corticosteroid	15 (38)
Topical nitrogen mustard	3 (8)
Topical bexarotene	2 (5)
Topical imiquimod	2 (5)
Phototherapy	4 (10)
Radiation therapy	1 (3)
Systemic therapy	9 (23)
Adjuvant bleach bath, n (%)	4 (10)

^1^Total percentages do not equal 100%, as some patients receive multiple treatment modalities.

Fourteen patients (36%) had sample collected in the trunk, 7 (18%) in the buttock, 14 (36%) in the extremities and 4 (10%) in the head and neck region. At the time of sample collection, 38 (97%) patients had patch/plaque-stage disease and 1 (3%) had tumor-stage disease. All the samples were obtained from the same type of ecological skin area, dry skin.

Twelve patients (31%) were newly diagnosed with MF and had not yet received therapy. Among the 27 patients receiving therapy, 19 patients received a topical treatment, 4 patients received phototherapy, 9 patients received systemic therapy, and 1 patient received radiation therapy. Topical corticosteroids were the most common topical therapy and were used in 15 patients. Four patients used bleach baths as adjuvant therapy.

### Bacterial Composition Changes in Lesional Skin and Non-Lesional Skin Among MF Patients

In total, 7, 597, 376 high-quality sequence reads were obtained (median per sample, 94,967 reads; range, 616-185, 930 reads), which belonged to 501 operational taxonomic units (OTUs) of 10 different bacterial phyla. Both positive and negative control has passed the internal quality control with Q30 score of 87% (threshold was > 65%).

**Figure 1:**
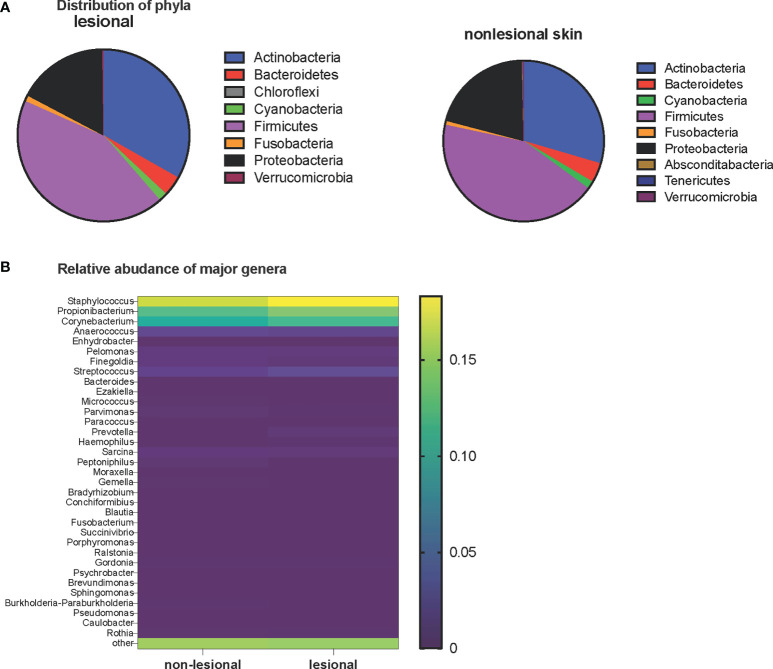
**(A)** Distribution of phyla in the lesional (left pie chart) and non-lesional (right pie chart) skin of patients with MF. **(B)** Relative abundance of major genera in non-lesional and lesional skin. Heatmap is based on the logit-transformed median relative abundance. The top of the yellow color gradient bar corresponds to a median relative abundance of 20%, and the bottom of the purple gradient bar corresponds to a median relative abundance of 0.

Firmicutes was the most predominant phyla, with a median relative abundance of 43% in both lesional and non-lesional skin. Other common phyla included Actinobacteria, Proteobacteria, and Bacteroidetes (**Figure 1A**). A comparison of the most abundant 34 genera between lesional and non-lesional skin is shown in **Figure 1B**. *Staphylococcus*, *Propionibacterium*, and *Corynebacterium* were the 3 most common genera, with a median relative abundance of 18.3%, 14.2%, and 11.8% for lesional skin and 16.7%, 12.5%, and 10.7% for non-lesional skin, respectively.

The relative abundance of 5 OTUs were significantly different between lesional and non-lesional skin. *Corynebacterium*-1, *Corynebacterium*-2, and *Neisseriaceae* were more prevalent in lesional skin. *Enhydrobacter* and *Sandaracinobacter* were more prevalent in non-lesional skin. On the OTU level, different members of the same species were identified that could not be further classified. OTUs were named by the species followed by a number (e.g., *Corynebacterium-1* and *Corynebacterium-2*) (**Figure S2**).

### Microbiota Diversity in Lesional and Non-Lesional Skin

**Figure 2:**
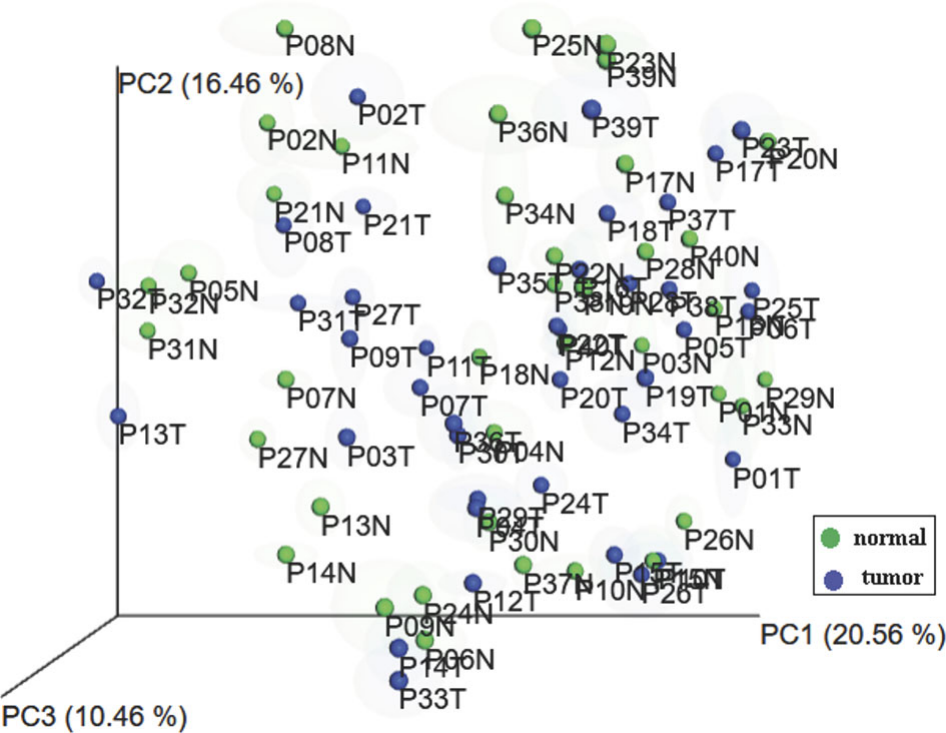
Principal coordinates analysis plots of microbial community composition. Samples from each patient cluster closer together rather than separating into lesional and non-lesional skin groups.

To evaluate whether there was a difference in skin microbiota composition between lesional and non-lesional skin, the beta diversity between samples was visualized (**Figure 2**). There was no statistically significant difference of beta diversity between the lesional and non-lesional skin. Principal coordinate analysis plots of Weighted UniFrac distance for beta diversity showed that interpersonal variability in skin microbiota is greater than the intrapersonal changes caused by cancer involvement. This observation potentially reflects that there are the global skin microbiota changes that are caused by MF instead of regional skin changes.

Non-lesional skin samples demonstrated a trend toward higher alpha diversity than lesional skin samples, although no significant difference was observed (*P* = .29) (**Figure S3**).

### Changes in Microbiota Composition Associated With Symptoms and Signs

To account for the variation between individuals, anatomical sites, and low biomass of the skin, we used the changes in the relative abundance of each genus from the contralateral non-lesional skin (control) to the lesional skin as a measure of skin microbiota changes related to the presence of MF, instead of directly comparing the skin microbiota of lesional skin to that of non-lesional skin as two groups (Nath et al., 2020). By using this method, we assumed that the skin microbiota of one area is the same as that of the contralateral area if disease is not present. To conduct the analyses for this pilot study with limited patients, we also assumed that the relative abundance changes of skin microbiota associated with the presence of MF were similar among different individuals at different anatomical locations.

#### Subjective Assessment

**Figure 3:**
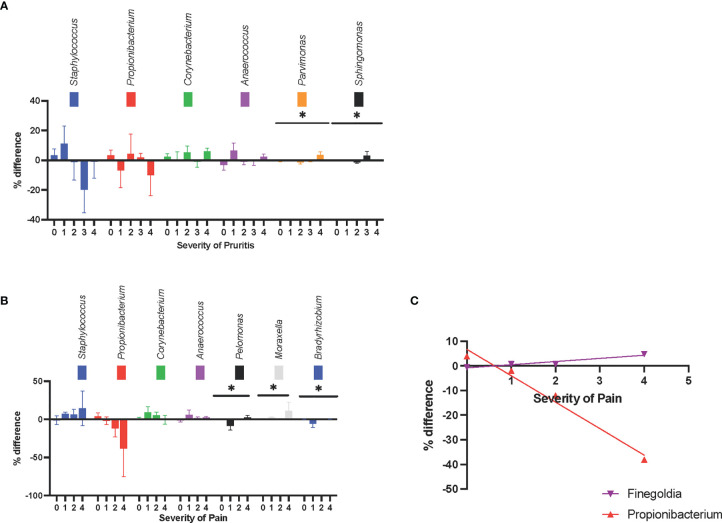
Symptom-associated changes in skin microbiota composition at the genus level. **(A)** The changes in relative abundance were shown at different pruritus level. **(B)** The changes in relative abundance were shown at different pruritus level, *Pelomonas* spp., *Moraxella* spp., and *Bradyrhizobium* spp. had statistically significant difference between pain levels. There was a trend that *Staphylococcus* spp. became more abundant and *Propionibacterium* spp. became less abundant, as the pain level increases. **(C)** The increase in the relative abundance of *Propionibacterium* spp. was negatively correlated with pain intensity. The increase in the relative abundance of *Finegoldia* spp. in lesional skin was positively correlated with pain intensity. *P ≤ 0.05.

At different pruritus levels (level 0-5, as scored by the patient), *Parvimonas spp* and *Sphingomonas spp* had statistically significant changes in relative abundance (*P* = .014 and *P* = .012, respectively) (**Figure 3A**).

At different pain levels (level 0-5, as scored by the patient), the changes in relative abundance of *Pelomonas spp*, *Moraxella spp*, and *Bradyrhizobium spp* were statistically significant (*P* = .028, *P = *.023, and *P = *.027, respectively). The relative abundance of *Pelomonas* and *Bradyrhizobium* were reduced in the lesional skin of patients with no or mild pain, and the relative abundance of *Moraxella* was increased in the lesions of patients with moderate or severe pain (**Figure 3B**). Although in-between group significance was not reached, the increase in the relative abundance of *Finegoldia spp* in lesional skin was positively correlated with pain intensity (r^2 ^= .91, *P* = .05), whereas the increase in relative abundance of *Propionibacterium* spp. was negatively correlated with pain intensity (r^2^ = .98, *P* = .01) (**Figure 3C**). The increase in relative abundance of *Staphylococcus* also showed a trend for positive correlation with pain intensity; however, this was not statistically significant (r^2^ = .89, *P* = .06).

#### Objective Assessment

**Figure 4:**
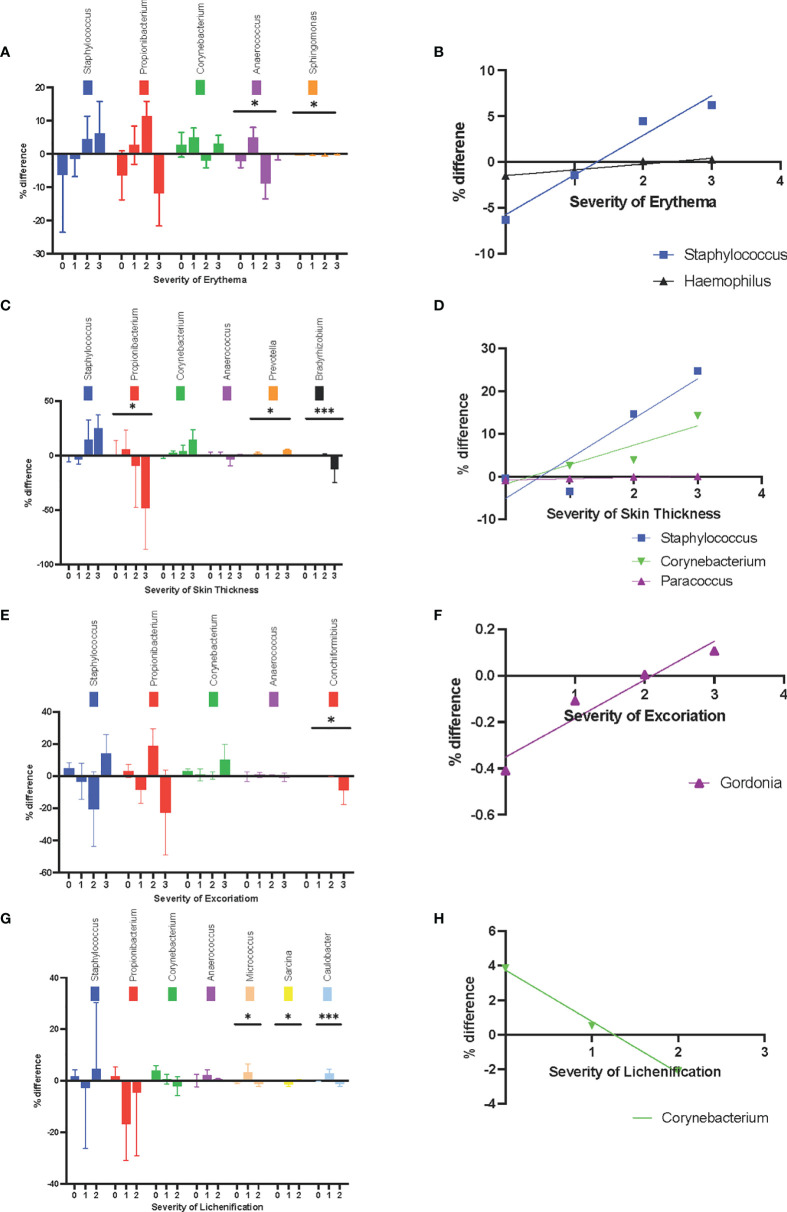
Sign-associated changes in skin microbiota composition at the genus level. **(A)** The changes in relative abundance at different erythema levels. **(B)** The increase in relative abundance of *Staphylococcus* spp. and *Haemophilus* spp. in lesional skin were positively corelated with erythema. **(C)** The changes in relative abundance at different skin thickness levels. **(D)** The increase in relative abundance of *Paracoccus* sp. in lesional skin was positively correlated with thicker skin. **(E)** The changes in relative abundance at different level of excoriation. **(F)** The increase in relative abundance of *Gordonia* spp. in the lesional skin was positively correlated with excoriation level. **(G)** changes in relative abundance at different levels of lichenification **(H)** The increase in relative abundance of *Corynebacterium* in lesional skin was negatively correlated with lichenification. *P ≤ 0.05; ***P ≤ 0.001.

At different erythema levels (level 0-3, as scored by a dermatologist), the changes in relative abundance of *Anaerococcus* spp. and *Sphingomonas* were statistically significant (*P* = .03 and *P* = .02, respectively) (**Figure 4A**). *Anaerococcus* was more common in non-lesional skin when the lesion was rated as level 2 or 3 for erythema. In the lesional skin with a higher degree of erythema, *Staphylococcus* spp. (r^2^_ _= .96, *P* = .02) and *Haemophilus* spp. (r^2 ^= .94, *P* = .03) were more common (**Figure 4B**).

At different skin thicknesses (level 0-3, as scored by a dermatologist), the changes in relative abundance of *Propionibacterium*, *Prevotella*, and *Bradyrhizobium* were statistically significant (*P *= .02, *P *= .03, and *P *= .0001, respectively) (**Figure 4C**). A decrease in the relative abundance of *Propionibacterium* and *Bradyrhizobium* in lesional skin was seen at higher levels of skin thickness. Conversely, *Paracoccus* sp. was more prevalent in lesional skin among patients with thicker lesions (r^2 ^= .93, *P* = .04) (**Figure 4D**).

At the highest level of excoriation signs (level 0-3, as scored by a dermatologist), the reduction in relative abundance of *Conchiformibus spp* was statistically significant (*P* = 0.004) (**Figure 4E**). The change in relative abundance of *Gordonia* spp. was positively correlated with the level of excoriation (r^2 ^= 0.92, *P* = .04) (**Figure 4F**).

At different levels of lichenification (level 0-3, as scored by a dermatologist), the change in relative abundance of *Micrococcus* spp.*, Sarcina* spp., and *Caulobacter* spp. were statistically significant (*P* = .04, *P* = .006, and *P* = .0009, respectively) (**Figure 4G**). Interestingly, *Corynebacterium* became less prevalent in lesions with higher levels of lichenification (r^2 ^= .99, *P* = .05) (**Figure 4H**).

#### Therapy Related Changes

Among 39 patients, 27 patients were on therapy (topical, systemic, radiation therapy or phototherapy) and 12 patients were not on any therapy. Compared to patients who were not on therapy, patients who were on any therapy had higher relative abundance of *Sarcina* spp. and lower relative abundance of *Sphingomonas* spp. in the lesional skin. The mean rank of the relative abundance difference of *Sarcina* spp. was 23.59 for the patients receiving any therapy and 11.92 for those receiving no therapy (P = .002). The mean rank of relative abundance difference of *Sphingomonas* spp. was 17.26 for patients receiving any therapy, and 26.17 for those receiving no therapy (P=.016).

Fifteen patients (38.5%) applied topical corticosteroid therapy within 24 hours of samples collection. There was no statistically significant difference in the relative abundance changes of any tested genus between the patients who received topical corticosteroid therapy and those who did not.

## Discussion

**Figure 5:**
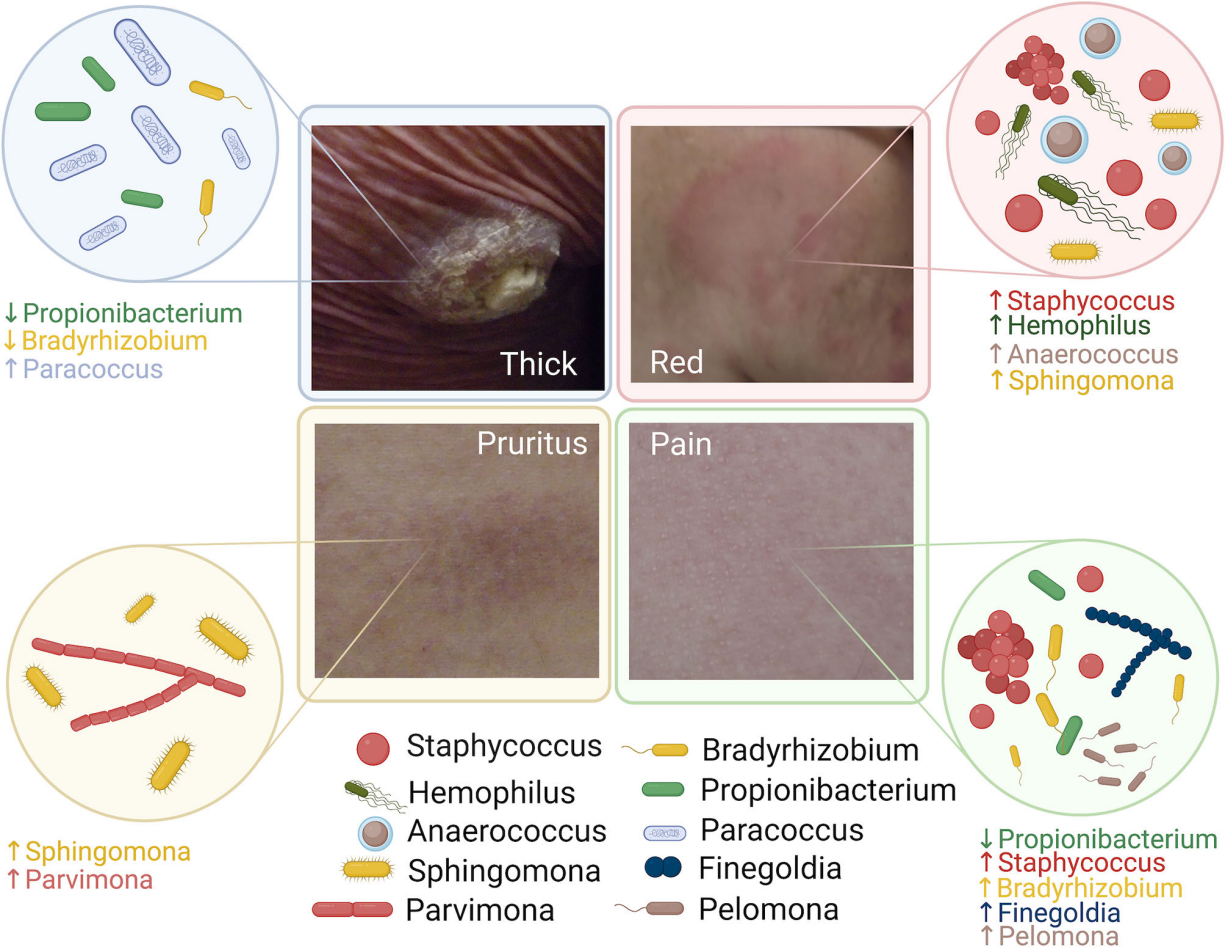
Summary of the microbiota alteration associated with disease phenotype in patients with Mycosis Fungoides.

In-depth profiling of cutaneous microbiota facilitated by high throughput 16S ribosomal RNA (rRNA) sequencing has improved our understanding of the roles of microbes in cancer development. However, limited studies have been specifically focused on the skin microbiota changes of patients with MF related to the disease phenotype. Here, we report our cohort of 39 patients with MF in a pilot cross-sectional study (**Figure 5**).

Compared to non-lesional skin, lesional skin showed a trend for less bacterial diversity and richness. However, statistical significance of this comparison was not reached, likely because of the small sample size of this pilot study and significant interpersonal variation. When examined at the OTU level, *Corynebacterium* and *Neisseriaceae* were more abundant in lesional skin, whereas *Sandaracinobacter* and *Enhydrobacter* were more abundant in non-lesional skin. The increase in abundance of *Corynebacterium* in lesional skin has also been reported by Harkin et al. (Harkins et al., 2020). A similar observation of increased *Corynebacterium* was previously described in atopic dermatitis and psoriasis (Zheng et al., 2019; Yerushalmi et al., 2019). Ridaura, et al. reported that C. accolens in the skin increased interleukin-23 production and activated a subset of γδ T cells, both of which promote skin inflammation (Ridaura et al., 2018).

In our study, we found distinct skin microbiota signatures associated with different clinical symptoms and signs. For example, thicker skin and higher pain levels significantly correlated with reduced relative abundance of *Propionibacterium* in lesional skin. This finding was also observed in atopic dermatitis (Bjerre et al., 2017). Whether *Propionibacterium* plays a protective role in reducing symptoms and inflammation or simply reflects microbial dysbiosis still remains to be answered*. Staphylococcus* was associated with more pronounced erythema and higher pain intensity among patients with MF. In a mouse model, *S. aureus* colonization has been found to cause strong T helper 17 cell polarization and drive inflammation (Chang et al., 2018). In addition, in patients with atopic dermatitis *S. aureus* skin colonization was associated with symptom severity (Chiu et al., 2009).

The remaining bacteria in our results are not well described in the skin microbiota literature. Among them, *Sphingomonas, Bradyrhizobium*, and *Gordonia* are ubiquitous organisms in the environment. Skin-resident gram-positive bacteria, such as *Parvimonas*, *Micrococcus*, *Finegoldia*, and *Anaerococcus*, occasionally cause skin infections and chronic wounds (Murphy and Frick, 2013, Smith et al., 1999). However, gram-negative bacteria, such as *Prevotella*, *Moraxella*, and *Neisseriaceae*, are not frequently found on the skin, but they can rarely cause skin infections (Mehmood et al., 2014; Liu et al., 2015; Rabionet et al., 2016). *Pelomonas*, *Sarcina*, and *Caulobacter* have not been previously described in skin infections.

To the best of our knowledge, this is the first study investigating the skin microbiota changes related to the disease phenotype of MF, though several studies have investigated the role of skin microbiota in its pathogenesis. Our study suggests that the skin microbiota signatures of MF share similar patterns with other non-malignant skin conditions such as atopic dermatitis and psoriasis (Chiu et al., 2009; Bjerre et al., 2017; Yerushalmi et al., 2019; Zheng et al., 2019). This raises the question whether the altered microbial composition directly influence the disease phenotype, contributes to the underlying pathogenesis of the skin disorders, or reflects the preferential selection of particular bacteria inherent to MF (Gilbert et al., 2018).

We also found that ongoing therapy can potentially alter certain skin microbiota signature such as *Sphingomonas* spp. and *Sarcina* spp. However, there was no statistically significant difference in skin microbiota signature between patients receiving topical corticosteroid therapy and those not receiving such therapy. The lack of the statistical significance with topical corticosteroid therapy could be from small sample size and lack of power. It could also potentially suggest that skin microbiota do not directly interact with a specific therapy, instead, they change due to the immune environment related to the cancer, which can be altered by various therapies.

Another unique feature of our study is that we did not compare the skin microbiota of the lesional skin and the non-lesional skin as two separate groups. The principal components analysis (PCA) using weighted Unifrac showed that the interpersonal variability was more significant than the changes related to the involvement of MF in the same individual. Therefore, we used the difference in the relative abundance of bacteria between lesional and non-lesional skin as a measure of the changes in skin microbiota during the development of MF, under the assumptions that skin microbiota should be the same in the opposite anatomic sites when the disease is not involved. This method facilitated the identification of the skin microbiota patterns despite small sample size under the assumption that the skin microbiota changes related to MF was similar among different individuals and anatomical area.

### Limitations of the Study

Our study has several limitations. First, though it represents one of the largest skin microbiota studies for patients with CTCL, the sample size was small. Second, because of technology limitations with 16s rRNA sequencing, the species identification can be inaccurate at times. Therefore, our study focuses on composition change at the genus level. Third, because of the small sample size, we did not have adequate power to assess the relationship between skin microbiota changes related to various anatomical area. Our study included both the patients on and off various therapies and was inadequate to evaluate the effect of different therapies on the skin microbiome. However, the analysis was done under the assumption that the changes in skin microbiota related to MF was similar among different individuals and anatomical area.

### Future Directions

Larger studies are needed to confirm our observations. Longitudinal studies can characterize the temporal skin microbiota changes at the time of disease diagnosis and then at a later timepoint when the patient is in remission.

## Conclusions

This pilot study described the regional changes in skin microbiota diversity and composition among 39 patients with MF. We identified microbial signatures associated with different symptoms, signs, and treatments. Findings in this study suggest a potential microbial role in changing skin phenotypes among patients with CTCL. However, larger studies are needed to elucidate the role of skin microbiota in disease pathogenesis and to evaluate the role of topical antibiotic therapies in mitigating symptoms and delaying disease progression.

## Methods

### Study Design and Patients

We performed a prospective pilot study of patients with MF treated at Moffitt Cancer Center from March, 2019 to June, 2019. The inclusion criteria were having biopsy-proven MF according to World Health Organization (WHO) European Organization for Research and Treatment of Cancer (EORTC) criteria (Willemze et al., 2019), being more than 18 years of age, having active skin lesions, and being able to answer English questionnaires. Patients who had signs of active skin infection (determined by evaluation of a board-certified dermatologist), used systemic antibiotics within 1 month of the study, used topical antibiotics within 1 week of the study, or showered within 12 hours of the study were excluded.

MF was staged at the time of diagnosis with tumor-node-metastasis-blood (TNMB) criteria based on International Society for Cutaneous Lymphomas (ISCL)/EORTC revised staging and classification guidelines (Olsen et al., 2007). The subjective assessment of symptom intensity was completed by patients *via* a 2-question questionnaire regarding the degree of pruritus and pain, the 2 most common and bothersome symptoms for MF (Wright et al., 2013; Semenov et al., 2020). Each symptom was rated by patients on a scale of 0 to 5, with 5 being the most intolerable. An objective assessment of lesion severity was completed by a dermatologist using 4 questions regarding the degree of lesion erythema, thickness, excoriation, and lichenification. Each sign was rated by the same dermatologist on a scale of 0 to 3 (none, mild, moderate, and severe). Photographs of the lesional and non-lesional sites were taken.

The study was approved by the Institutional Review Board at the University of South Florida (MCC No. 19854), and written informed consent was obtained from all patients.

### Microbial Sample Collections

Skin microbial samples were taken from the lesional skin and the contralateral non-lesional healthy skin. Samples were collected by the same person using sterile gloves and according to a standardized procedure (SanMiguel et al., 2018). The skin surface was swabbed with a sterile swab (BD BBL Culture Swab II; Becton, Dickinson and Company [BD], Franklin Lakes, NJ) moistened with sterile NaCl 0.9%. After collection, the samples were frozen at -80 °C until further processing.

### DNA Isolation and 16S Ribosomal RNA Sequencing

DNA was extracted from the samples using the Qiagen MagAttract PowerSoil DNA KF Kit (Qiagen, Hilden, Germany) optimized for the ThermoFisher KingFisher robot (Thermo Fisher Scientific, Waltham, MA). Both negative controls and positive controls were used. The positive controlled consisted of cloned Thioglobaceae SUP05, a Gamma-Proteobacteria; while negative control was extraction control negative, template-free DNA. The kit used magnetic beads to capture DNA while excluding organic inhibitors specifically. Qubit (Thermo Fisher Scientific) was used for DNA quantification and quality check. The microbial composition of samples was characterized by sequencing the V4 hypervariable region of the 16S rRNA gene using the Illumina MiSeq sequencer (Illumina, San Diego, CA) (Meisel et al., 2016). Samples that failed PCR or had spurious bands were re-amplified by optimizing PCR conditions. We used a high-throughput SequalPrep96-well plate kit (Thermo Fisher Scientific) to clean up and normalize PCR reactions.

### Bioinformatics Processing

To remove adaptors and low-quality reads, paired-end reads were cleaned using Trimmomatic (USADELLAB.org) (Bolger and Giorgi, 2014) with the following parameters: LEADING:10 TRAILING:10 SLIDINGWINDOW:4:15 MINLEN:40. Treatment samples with a minimum of 2000 reads were kept for further analyses. After this, the chimeric reads were searched against the 16S rRNA Gold database with the default UCHIME (4.2) parameters (Edgar, 2016). Next, the cleaned reads were merged with Paired-End reAd mergeR (PEAR; 0.9.10; The Exelixis Lab, Heidelberg, Germany) (Zhang et al., 2014). Finally, OTUs were generated by open reference of the Quantitative Insights Into Microbial Ecology (QIIME 1.9.1) pipeline (Caporaso et al., 2010). The database used for taxonomic assignment was Silva 128 97_otus_16S.fasta (Quast et al., 2013). Only OTUs with the minimum of 312 total observation count were retained. To characterize a microbial community, alpha and beta diversities were also analyzed using QIIME 1.9.1.

### Statistical Analyses

The relative abundance of OTUs was compared between lesional skin and matched non-lesional skin using paired *t* tests. Two-sided *P* < 0.05 and absolute fold change of >1.5 were considered statistically significant. To characterize richness of the microbiome, genus- and phylum-relative abundance were visualized using pie charts. Alpha diversity was measured using Shannon entropy and compared using the Wilcoxon signed rank test. The beta diversity between samples was visualized with a principal components analysis (PCA) using weighted Unifrac distance on centered log-ratio–transformed featureCount data.

We calculated the difference in the genus-relative abundance in skin microbiota between lesional and non-lesional skin to reflect the changes in skin microbiota. A positive value indicated higher relative abundance of a genus in lesional skin than in non-lesional skin, and a negative value indicated higher relative abundance of a genus in non-lesional skin. One-way analysis of variance (ANOVA) tests were used to evaluate the difference in skin microbiota changes between different levels of symptoms, and signs. Pearson product-moment correlations were used to determine how strong the relationship was between the degree of symptoms and the changes in abundance of a specific genus. Linear regression models were used to assess the magnitude of change in relative abundance based on changes in the symptoms/signs. When statistical significance was found, the data were then fitted using a simple linear regression model. Wilcoxon rank-sum test was used to evaluate the difference in skin microbiota changes between therapy and no therapy group.

Statistical analyses and representations were performed using the GraphPad Prism software (**GraphPad** Software, San Diego, CA) using statistical packages in R.

## Data Availability Statement 

The datasets presented in this study can be found in online repositories. The names of the repository/repositories and accession number(s) can be found below: https://www.ncbi.nlm.nih.gov/geo/query/acc.cgi?acc=GSE186083.

## Ethics Statement

The studies involving human participants were reviewed and approved by the University of South Florida IRB, Protocol No. MCC19854. The patients/participants provided their written informed consent to participate in this study.

## Author Contributions

YZ: Conceptualization, Formal Analysis, Funding acquisition, Investigation, Writing – original draft, Visualization. LS-V: Investigation, Resources, Writing – review and editing. LC: Investigation. MH: Resource. JY: formal analysis, visualization. DR: Visualization, writing – review and editing. YK: methodology, formal analysis, visualization. AG: Supervision, Funding acquisition, resources, writing – review and editing; LR: writing-review and editing, resources; LS: Conceptualization, supervision, writing-review and editing. All authors contributed to the article and approved the submitted version.

## Funding

This work was supported through NIH/NCI grant titled Infection and Cancer Research: A New Frontier in Cancer Prevention and Control (5K05CA181320) and a grant received from the University of South Florida, Graduate Medical Education Office.

## Conflict of Interest

The authors declare that the research was conducted in the absence of any commercial or financial relationships that could be construed as a potential conflict of interest.

## Publisher’s Note

All claims expressed in this article are solely those of the authors and do not necessarily represent those of their affiliated organizations, or those of the publisher, the editors and the reviewers. Any product that may be evaluated in this article, or claim that may be made by its manufacturer, is not guaranteed or endorsed by the publisher.

## Supplementary Material

Supplementary Figure 1 Deidentified photograph of the skin lesions from 39 included patients at the time of sample collection. The involved total body surface area % and mSWAT were annotated on the graph.

Supplementary Figure 2 Comparative analysis of the OTU specific relative abundance of *Corynebacterium*-1, *Corynebacterium*-2, *Neisseriaceae*, *Enhydrobacter*, and *Sandaracinobacter* between lesional and non-lesional skin. Each dot represents the average relative abundance of the microbiota in lesional and non-lesional skin from each participant. Box represented median and 1st/3rd quartile values (the top 25% and 75% values).

Supplementary Figure 3 Comparison of alpha diversity (Shannon entropy) between lesional and non-lesional skin samples. Alpha diversity measures diversity and richness between lesional and non-lesional skin samples. The lesional skin samples had increased alpha diversity. However, this was not statistically significant. Boxes represent median and 1st/3rd quartile values (the top 25% and 75% values).

## Abbreviations

ANOVA, analysis of variance; CTCL, Cutaneous T cell lymphoma; EORTC, European Organization for Research and Treatment of Cancer; ISCL, International Society for Cutaneous Lymphomas; MF, Mycosis fungoides; OTUs, operational taxonomic units; PEAR, Paired-End reAd merger; QIIME, Quantitative Insights Into Microbial Ecology; rRNA, ribosomal RNA; TNMB, tumor-node-metastasis-blood; WHO, World Health Organization.
