# Supplementary figures and images for: “Alterations in the Skin Microbiota Are Associated With Symptom Severity in Mycosis Fungoides”
Source: Front Cell Infect Microbiol. 2022 May 17;12:850509. doi: 10.3389/fcimb.2022.850509 (PMC9152451; doi:10.3389/fcimb.2022.850509)

Figure S1

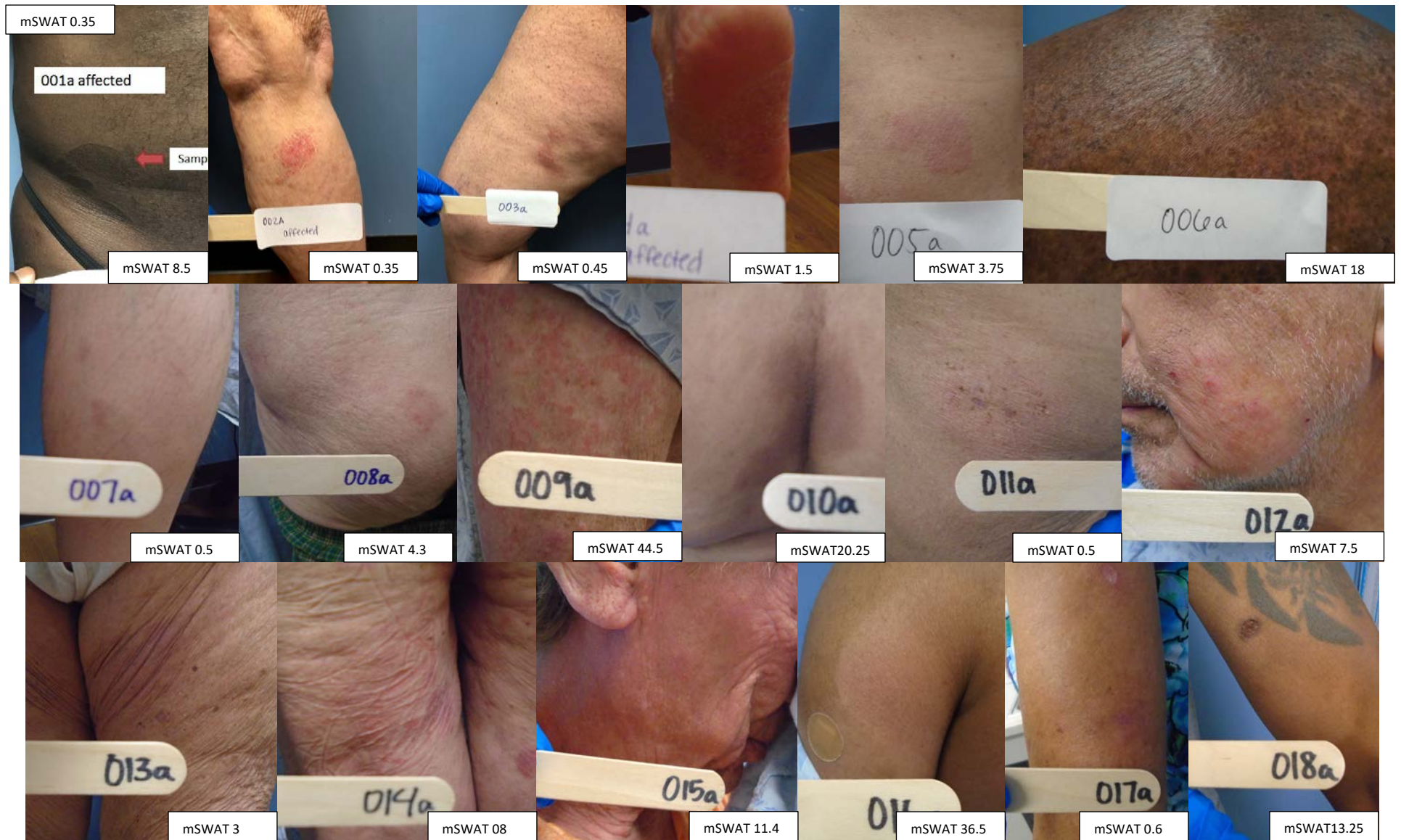

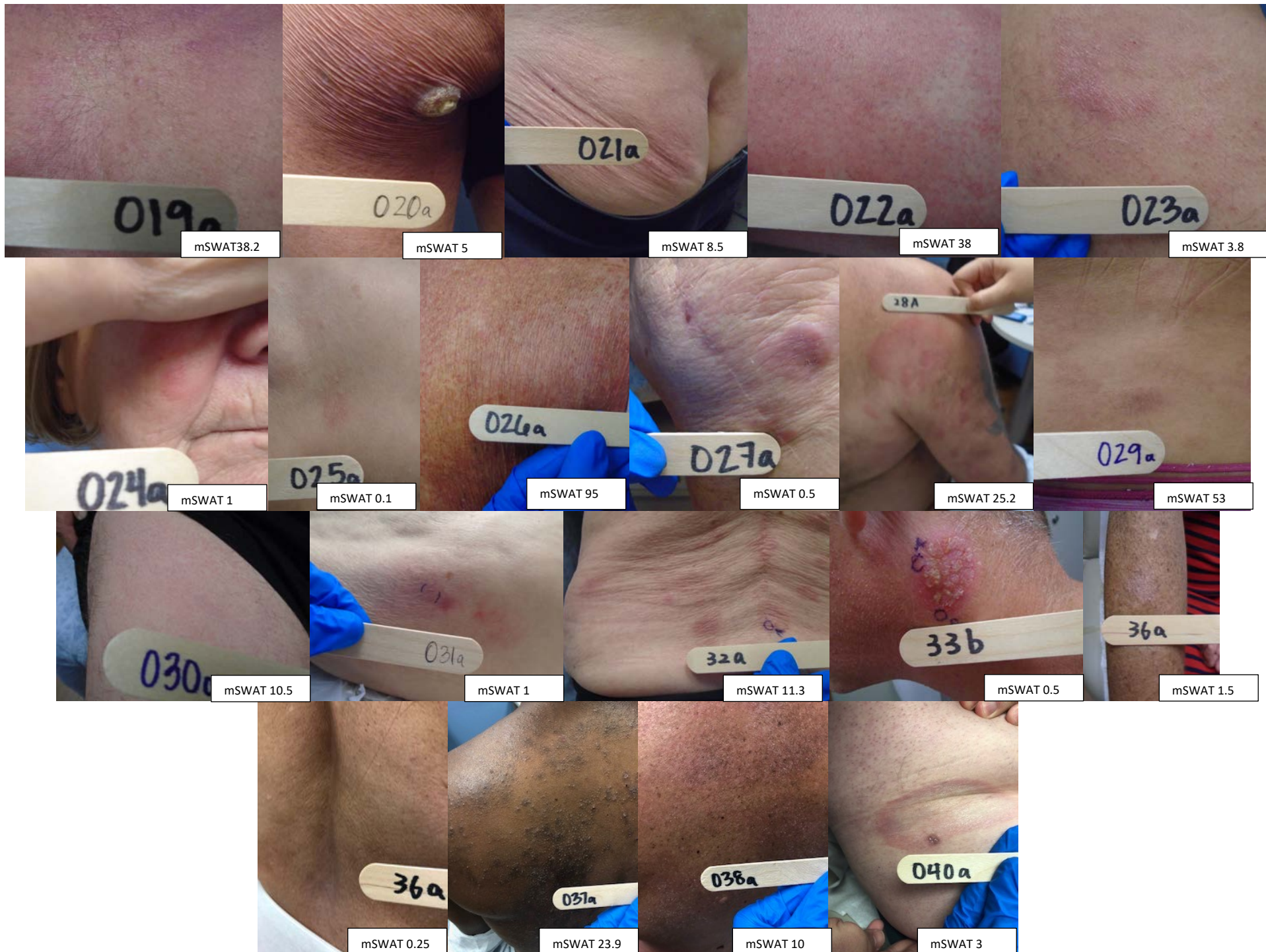

Supplement: Supplementary Figure 1 — Deidentified photograph of the skin lesions from 39 included patients at the time of sample collection. The involved total body surface area % and mSWAT were annotated on the graph. [file DataSheet_1.pdf]

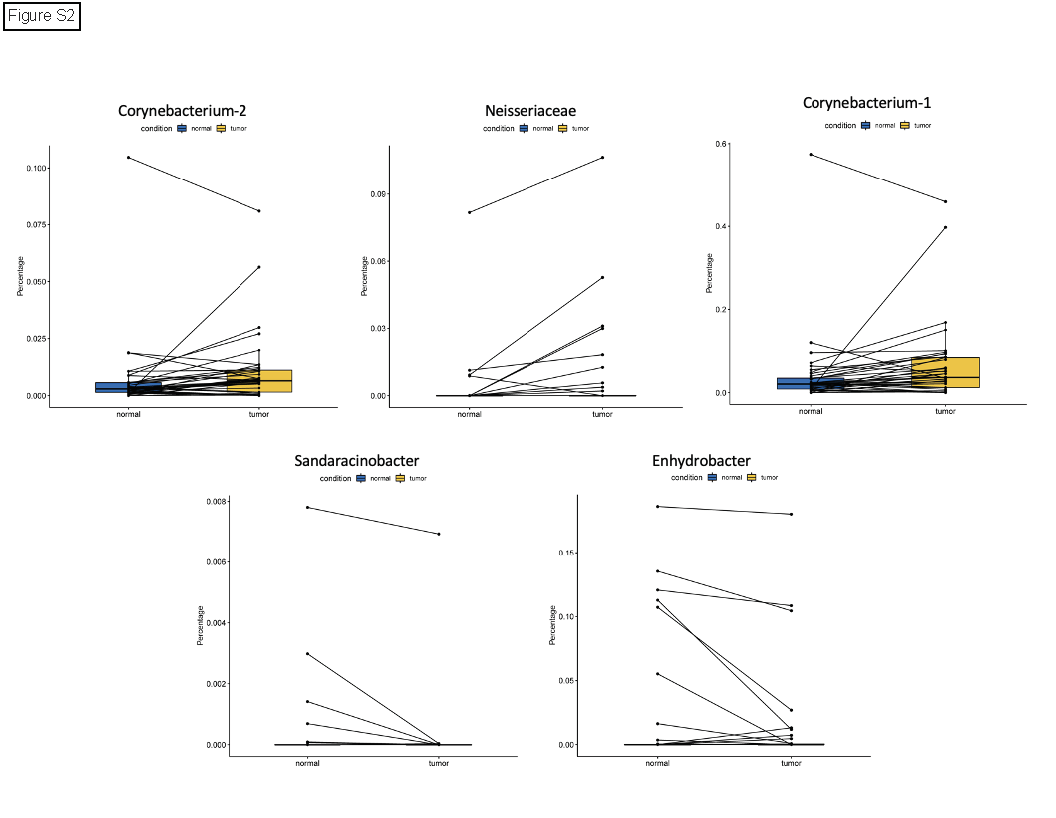

Supplement: Supplementary Figure 2 — Comparative analysis of the OTU specific relative abundance of Corynebacterium-1, Corynebacterium-2, Neisseriaceae, Enhydrobacter, and Sandaracinobacter between lesional and non-lesional skin. Each dot represents the average relative abundance of the microbiota in lesional and non-lesional skin from each participant. Box represented median and 1st/3rd quartile values (the top 25% and 75% values). [file Image_1.tiff]

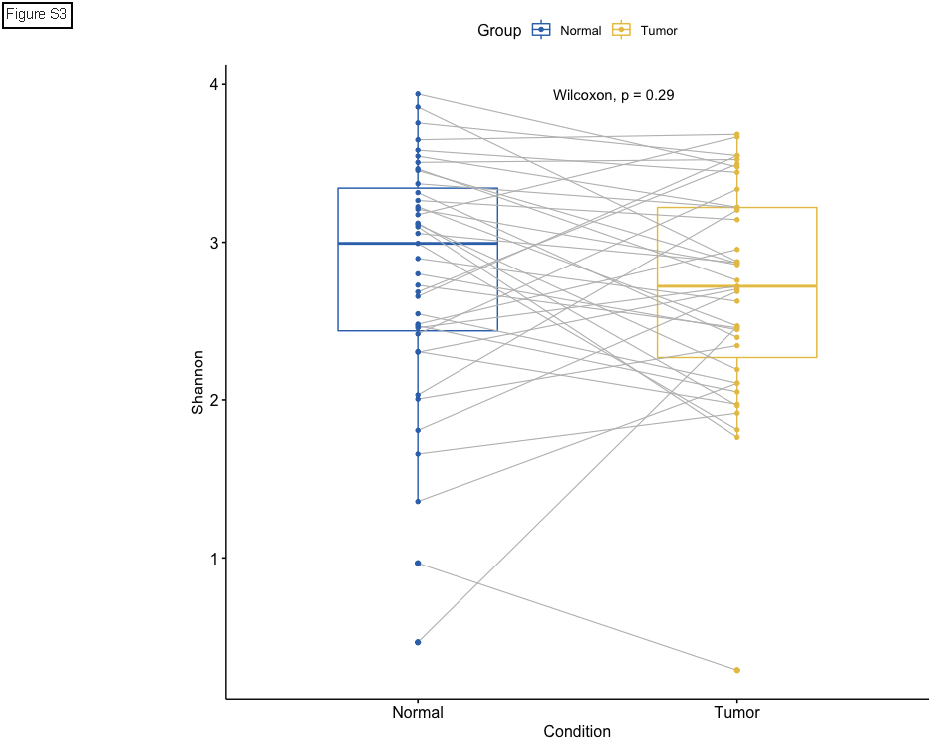

Supplement: Supplementary Figure 3 — Comparison of alpha diversity (Shannon entropy) between lesional and non-lesional skin samples. Alpha diversity measures diversity and richness between lesional and non-lesional skin samples. The lesional skin samples had increased alpha diversity. However, this was not statistically significant. Boxes represent median and 1st/3rd quartile values (the top 25% and 75% values). [file Image_2.tiff]
